# Bioinspired, vertically stacked, and perovskite nanocrystal–enhanced CMOS imaging sensors for resolving UV spectral signatures

**DOI:** 10.1126/sciadv.adk3860

**Published:** 2023-11-03

**Authors:** Cheng Chen, Ziwen Wang, Jiajing Wu, Zhengtao Deng, Tao Zhang, Zhongmin Zhu, Yifei Jin, Benjamin Lew, Indrajit Srivastava, Zuodong Liang, Shuming Nie, Viktor Gruev

**Affiliations:** ^1^ Department of Engineering and Computer Engineering, University of Illinois at Urbana-Champaign, 306 N Wright St, Urbana, IL 61801, USA.; ^2^ Department of Bioengineering, University of Illinois at Urbana-Champaign, 1406 W Green St, Urbana, IL 61801, USA.; ^3^ College of Engineering and Applied Sciences, Nanjing University, 163 XianLin Ave, Nanjing, Jiangsu 210023, China.; ^4^ School of Chemistry and Chemical Engineering, Yangzhou University, 180 Siwangting Road, Yangzhou, Jiangsu 250002, China.; ^5^ Beckman Institute for Advanced Science and Technology, University of Illinois at Urbana-Champaign, 405 N Mathews Ave, Urbana, IL 61801, USA.; ^6^ Carle Illinois College of Medicine, University of Illinois at Urbana-Champaign, 506 South Mathews Ave, Urbana, IL 61801, USA.

## Abstract

Imaging and identifying target signatures and biomedical markers in the ultraviolet (UV) spectrum is broadly important to medical imaging, military target tracking, remote sensing, and industrial automation. However, current silicon-based imaging sensors are fundamentally limited because of the rapid absorption and attenuation of UV light, hindering their ability to resolve UV spectral signatures. Here, we present a bioinspired imaging sensor capable of wavelength-resolved imaging in the UV range. Inspired by the UV-sensitive visual system of the *Papilio xuthus* butterfly, the sensor monolithically combines vertically stacked photodiodes and perovskite nanocrystals. This imaging design combines two complementary UV detection mechanisms: The nanocrystal layer converts a portion of UV signals into visible fluorescence, detected by the photodiode array, while the remaining UV light is detected by the top photodiode. Our label-free UV fluorescence imaging data from aromatic amino acids and cancer/normal cells enables real-time differentiation of these biomedical materials with 99% confidence.

## Teaser

*Papilio xuthus* butterfly–inspired camera with perovskite nanocrystals enables cancer cell identification via UV autofluorescence.

## INTRODUCTION

The compound eyes of butterflies present a fascinating contrast to the standard trichromatic vision of human eyes. Unlike humans, butterflies have compound eyes with six or more photoreceptor classes, each with distinct spectral sensitivities in their optical sensor units, known as ommatidia (*1*–*3*). This remarkable feature allows butterflies to perceive a broader range of colors and details in their visual environment. Among these fascinating creatures, the *Papilio xuthus*, a yellow-colored swallowtail butterfly of medium to large size, stands out with its tetrachromatic vision. The eyes of the *P. xuthus* house ultraviolet (UV), violet, blue, green, red, and broadband receptors, enabling it to detect UV light and discern subtle variations in spectral composition.

**Fig. 1.**
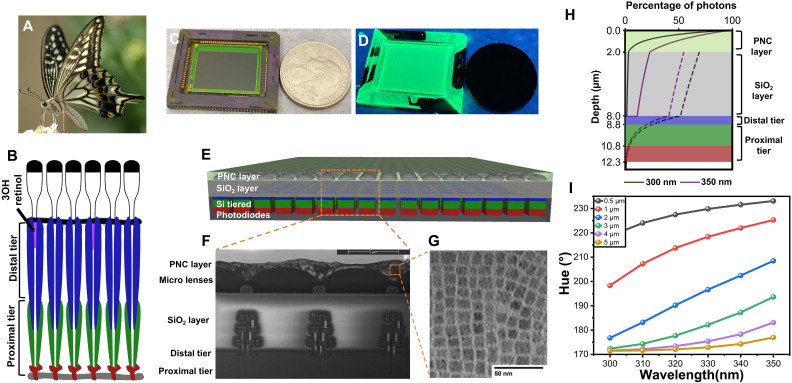
Schematic diagram of the *P. xuthus* butterfly–inspired UV sensor with PNCs. (**A**) Our UV imaging sensor is inspired by the *P. xuthus* butterfly’s visual system. (**B**) Butterfly’s ommatidia, featuring tiered photodetectors and fluorescent pigments for wavelength resolved UV sensing. (**C**) Bioinspired sensor under white light. (**D**) Green appearance of the sensor under UV light, attributed to perovskite nanocrystal (PNC) layer fluorescence. (**E**) Block diagram showing PNC layer and vertically stacked photodiodes. (**F**) Cross-sectional SEM image of the PNC-coated sensor. (**G**) Transmission electron microscopy (TEM) image of 13-nm cubic PNCs. (**H**) Simulations showing UVB detection by PNC and UVA detection by both PNC and silicon photodetectors. (**I**) Optimization of PNC thickness for maximum UV target hue separation, achieved at 2 μm. Photo credit: Wikimedia Commons/Laitche.

Notably, the ommatidia of the *P. xuthus* butterfly exhibit a tiered structure in their photoreceptors, with distal and proximal receptors having distinct spectral sensitivities (Fig. 1, A and B). The sensitivity to UV light is achieved through two different mechanisms used by separate sets of photoreceptors. In approximately one-third of the ommatidia, a thin layer of UV fluorescent pigments, such as 3-hydroxyretinol or 3OH retinol, is located near the distal part of the ommatidium. When UV photons reach these ommatidia, the pigments emit fluorescence, which is subsequently absorbed by the proximal photoreceptors with peak sensitivity in the green spectrum. In other regions of the ommatidia, distal photoreceptors directly absorb and detect UV photons. Because of the differing UV sensitivities between these two types of photoreceptors, the *P. xuthus* is capable of discriminating between targets with minimal variations in the UV spectrum, as small as 10 nm. Furthermore, the proximal photoreceptors detect longer-wavelength photons, including yellow and red, with the assistance of corresponding visual pigments, thus expanding the butterfly’s perception of its surroundings.

To replicate the UV sensing mechanisms and visible photon discrimination observed in the *P. xuthus* butterfly, we have successfully emulated these processes by combining a thin layer of metal-halide perovskite nanocrystals (PNCs) with a vertically tiered array of three silicon photodiodes (refer to Fig. 1, C to G). While various types of fluorescent nanocrystals, such as semiconductor quantum dots, can be used as down-conversion materials for converting UV light into visible fluorescence (*4*), we specifically opted for colloidal PNCs due to their exceptional structural, electronic, and optical properties (*5*–*7*). The colloidal PNCs exhibit remarkable attributes, including high defect tolerance, a high fluorescence quantum yield, a narrow emission linewidth, rapid radiative decay (short excited-state lifetime), and cost-effectiveness in large-scale synthesis and device fabrication (*8*–*12*). These fundamental characteristics have paved the way for exciting advancements in the field, such as the development of light-emitting diodes (LEDs) (*13*–*15*), ultraefficient solar conversion devices (*16*–*18*), broadband photodetectors (*19*, *20*), low-cost radiation scintillators (*21*–*23*), and color-enhanced optoelectronic displays (*24*, *25*).

In this study, we leverage the CsPbBr_3_ PNC layer, which efficiently absorbs UV photons within the UVB spectrum—specifically those with wavelengths exceeding 250 nm—and subsequently reemits fluorescent light in the green spectrum. This emitted light is subsequently detected by the underlying silicon photodiodes. Furthermore, the distal photodiodes exhibit sensitivity toward UV photons with wavelengths greater than 300 nm (primarily within the UVA spectrum) by virtue of their direct absorption and conversion into electron-hole pairs.

Contrary to traditional methods that use pigments to segregate UVA and visible photons of varying energies, the stratified photodiodes in this study exploit the inherent wavelength-dependent absorption coefficient present in silicon. Here, photons of shorter wavelengths, including UVA and blue light, demonstrate absorption coefficients an order of magnitude greater compared to those exhibited by photons in the red spectrum, i.e., those of longer wavelengths (*26*). As a consequence, the bulk of UVA and blue photons are absorbed in the distal photodiode, while red photons traverse across all tiers of photodiodes, with a substantial portion being absorbed in the proximal photodiodes, as shown in Fig. 1H. Given that PNCs exhibit minimal absorption in the visible spectrum, our imaging device is uniquely capable of capturing color information within a singular pixel. This capability is attributable to the wavelength-dependent photon absorption exhibited across the differing tiers of photodiodes.

## RESULTS

### PNC optimization for wavelength-resolved UV sensing

To elucidate the wavelength-resolved imaging capabilities of our sensor, along with its design optimization for discriminating UV signatures, we examine the mechanisms of photon absorption and photocurrent generation in response to two distinct sets of monochromatic photon fluxes within the UV spectrum, as illustrated in Fig. 1H. Let us begin by evaluating the impact of a photon flux within the UVA spectrum with a wavelength of 350 nm as it impinges upon a pixel of our device. The fraction of UVA photons absorbed varies on the basis of the thickness of the PNC layer; one portion of the photon flux is absorbed by the PNC layer, and another fraction is absorbed by the imager’s silicon dioxide layer as well as the distal silicon photodiodes. The absorption process for UVA photons is represented by a solid purple line in Fig. 1H.

The silicon dioxide layer, an integral component of imaging sensors, serves to isolate the metallic wires used to establish connections between transistors and photodiodes in the sensor. However, photons absorbed by this silicon dioxide layer do not contribute to the photogeneration process. Therefore, the thickness of this layer ought to be minimized or, alternatively, backside-illuminated technology could be used to augment sensitivity in the UV spectrum. The absorption process of photons within the PNC layer, silicon dioxide layer, and silicon photodiodes follows an exponential decay characteristic with respect to the photon propagation distance. This behavior is mathematically encapsulated by the equation *N* = *N*_0_*e*^−α*d*^, where *N* is the remaining photon flux after travelling distance *d*, *N*_0_ is the initial photon flux, and α is the absorption coefficient. The absorption coefficients for silicon dioxide and silicon are referred to in previous works (*27*, *28*).

The absorption of UVA photons within the PNC layer results in the emission of green, fluorescent photons in all directions, with a subset of these photons being absorbed by the underlying photodiodes. There are three key factors contributing to the observed losses in the final photogenerated electron-hole pairs in the silicon photodiodes. Initially, losses occur within the PNC layer due to defect states (*29*), which yield a photoluminescent quantum yield (PLQY) of approximately 80%. Subsequently, the isotropic emission of fluorescence within the PNC layer causes half of the green photons to radiate away from the photodiodes. Nevertheless, these photons undergo multiple reflections between the PNC layer’s top and bottom surfaces. The quantity of photons directed toward the underlying photodiodes can be estimated via a mathematical model proposed in earlier literature (*30*). As per these two forms of loss, about 70% of the initial photon count will remain as green, fluorescent photons reach the silicon layer, thus triggering the generation of electron-hole pairs within the underlying photodiodes. The absorption of these green, fluorescent photons by the silicon photodiodes is denoted by the dashed purple line in Fig. 1H. Last, a portion of the photogenerated electron-hole pairs will recombine, leading to no contribution to the final photocurrent.

Subsequently, we shift our focus to the photon absorption and photocurrent generation mechanisms in response to a UVB photon flux with a wavelength of 300 nm striking a pixel of our sensor. The distal photodiode lacks sensitivity toward UVB photons as they are wholly absorbed by the silicon dioxide layer. The PNC layer, however, absorbs UVB photons (represented by the solid black line in Fig. 1H), converts them into green photons (indicated by the dashed black line in Fig. 1H), which are then registered by the underlying photodiodes.

In summary, the top photodiodes accrue photogenerated charges owing to the absorption of both UVA photons and green photons fluorescently emitted from the PNCs, stimulated by both UVA and UVB photons. The middle and bottom photodiodes solely carry photogenerated charges as a result of the fluorescence emitted by the PNCs. The ratio of photogenerated charges across the three photodiodes will hinge on the intensity and spectral composition of the UV photons.

Last, to maximize the discernment of diverse UV wavelengths, the thickness of the PNC layer was optimized (see Materials and Methods and fig. S1). On the basis of the simulation results depicted in Fig. 1I, the optimal PNC layer thickness for the sensor was established to be approximately 2 μm. This precise thickness allows the sensor to display the broadest gamut of hues across the UV spectrum, spanning from 300 to 400 nm. Increasing the PNC layer thickness enhances UV sensitivity for both the UVA and UVB spectra but compromises the distinction of UV spectral signatures and leads to heightened cross-talk between pixels. This amplified optical cross-talk could blur the image and impair detection capabilities at the target edges, which hold substantial importance for margin-free cancer surgeries.

### Bioinspired UV sensor fabrication

The imaging sensor with vertically stacked photodiodes is fabricated by a standard semiconductor foundry. All-inorganic CsPbBr_3_ PNCs are synthesized by the hot injection method in vacuum at 180°C (*31*). The as-synthesized PNCs are then dissolved in hexane and spin coated at 1500 rpm on the imaging sensor’s surface (see Materials and Methods). The imaging device is retained on a heating plate at 50°C under a fume hood for another 15 min to ensure complete evaporation of hexane. The imaging sensor is packaged in a ceramic package and housed on a custom printed circuit board that houses multiple auxiliary electronics, such as 12-bit analog-to-digital converters, voltage regulators, and field programmable gate arrays for finite state machine implementation.

The final resolution of the imaging sensor is 2688 by 1792 pixels with 7.8-μm-pixel pitch. A cross-sectional scanning electron microscopy (SEM) profile of the sensor is shown in Fig. 1F. The PNC layer is coated on top of the microlenses, which focus the incident UV photons and fluorescence photons onto the underlaying photodiodes and minimize optical cross-talk. Figure 1G shows the transmission electron microscopy (TEM) image of the PNCs, which are stacked in cubic form with a side length of about 13 nm.

### Optoelectronic benchmarks

**Fig. 2.**
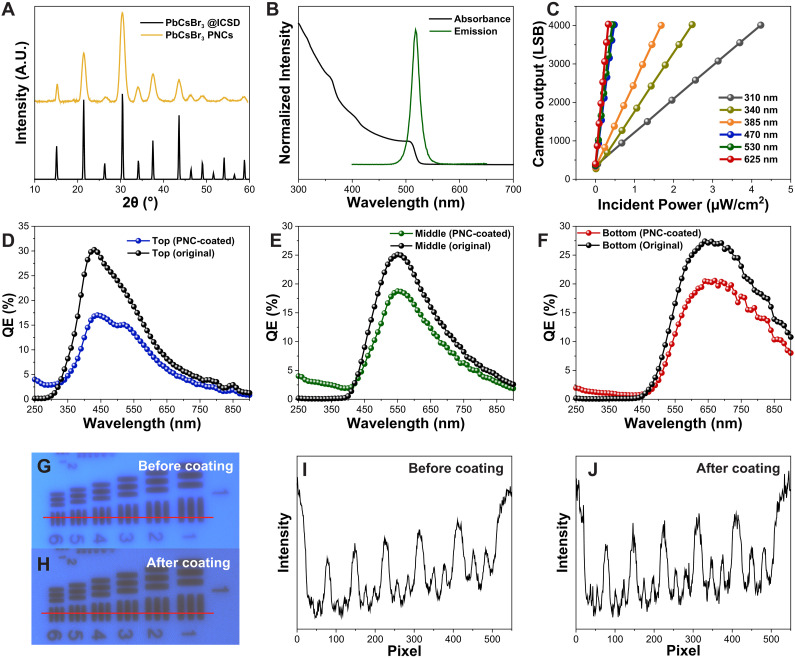
Optoelectronic analysis of our UV imaging sensor. (**A**) X-ray diffraction (XRD) pattern comparison of CsPbBr_3_ PNCs to the standard cubic phase. A.U., arbitrary units. (**B**) Absorption and emission spectra of PNC in hexane, both peaking at 518 nm. (**C**) Sensor’s digital output shows linear relation with incident optical power in UV and visible spectra. LSB, last significant bit. (**D** to **F**) Quantum efficiencies (QEs) of photodiodes, with notable UV enhancement in PNC-coated sensors. (**G** to **J**) Spatial resolution and digital output comparisons at 340 nm, showing that PNC layer does not affect UV resolution.

We conducted several benchtop tests to quantify the optoelectronic performance of the sensor. Because the PNC layer is essential for UV spectral imaging, we first validated the crystal lattice structure of the as-synthesized PNCs with x-ray diffraction (XRD) patterns (Fig. 2A). The XRD pattern of the PNCs is fully aligned with the Inorganic Crystal Structure Database reference standard for cubic CsPbBr_3_ crystals, granting our PNCs desirable semiconducting properties in cubic phase (*6*). Second, we evaluated the absorbance and photoluminescence (PL) emission spectra of the PNCs (Fig. 2B). The PNCs have a sharp emission peak at 518 nm with a full width at half maximum of 17 nm. The absorbance of the PNCs decreases with longer wavelengths and drops to zero at 518 nm, which is the onset of the fluorescence emission spectrum. Figure 1 (C and D) shows the imaging sensor under white light and UV illumination, respectively, compared to the size of a quarter. Bright green light can be observed from the sensor’s surface when illuminated with UV light due to the PNCs’ fluorescence. As discussed above, a portion of the fluorescent photons from the PNC layer will be emitted away from the sensor (*30*), resulting in a green surface coloration.

Next, we evaluated the linearity of the imager photo response for different incident optical powers at various wavelengths (Fig. 2C). The sensor’s linearity was evaluated in both the UV (310, 340, and 385 nm) and visible (470, 530, and 625 nm) spectrums. The incident optical power was swept from 0.04 to 4 μW/cm^2^, and the exposure time was adjusted for each wavelength to cover most of the dynamic range of the sensor. Despite the complex nonlinear photogeneration mechanisms of our imaging sensor described above, the photo response for our imager in both the UV and visible spectrums is above 99% linear over the imager’s dynamic range.

Spatial nonuniformity or fixed pattern noise (FPN) is another important metric that quantifies variations between the pixels’ output across the sensor when stimulated with the same photon flux. Because of additional fabrication steps required to realize our imaging sensor, the spatial nonuniformity is adversely affected. The FPNs for the imaging sensor before applying the PNC layer are 0.3% for the three photodiodes. After spin coating the PNC layer, the FPNs are increased to 1.7, 0.9, and 1.0% for the top, middle, and bottom photodiodes, respectively (fig. S2). The variations in the PNC layer thickness, which can be observed in the SEM image in Fig. 1F, are the main contributor to the increased nonuniformity. Because the top surface of the imaging sensor is not flat due to the existence of microlenses, the spin coating of the hexane-suspended PNCs will result in thickness variations across the imager. These thickness variations will lead to variations in both photon absorption and fluorescence emission intensity, which can be calibrated offline via per-pixel gain correction.

Although the PNC layer decreases the imager uniformity, it also improves quantum efficiency (QE) in the UV spectrum and enables UV spectral imaging without degrading spatial resolution. The QEs before and after spin coating the PNC layer for the top, middle, and bottom photodiodes are depicted in Fig. 2 (D to F), respectively. The top photodiode of the uncoated image sensor is sensitive to UVA photons ranging from 300 to 400 nm, as depicted in Fig. 2D. The QEs from 250 to 300 nm are enhanced by about 11, 30, and 15 times for the top, middle, and bottom photodiodes, respectively. Furthermore, the middle and bottom photodiodes have over 10 times higher QE for wavelengths between 300 and 400 nm with the PNC layer compared to without it. The top photodiodes do not have higher QE in 300- to 400-nm spectral range by design because of the limited PNC thickness necessary for UV spectral discrimination. In the visible spectrum (i.e., 400 to 700 nm), the PNC-coated imager has lower QE than the bare imager. This could be attributed to absorption by the PNC layer as well as change in the index of refraction surrounding the microlens arrays, which reduces the microlens’ photon redirection capabilities toward the photodiode area.

To evaluate the effects of the PNC layer on the imager spatial resolution, we imaged a 1951 U.S. Air Force (USAF) resolution test chart illuminated with a 340-nm LED light source with both bare and PNC-coated sensor. Intensity images recorded without and with the PNC layer are displayed in Fig. 2 (G and H), respectively. Both images appear to retain high-frequency features without any observable differences. To further examine the effects on spatial resolution, we plotted the digital pixel output across one row of pixels, denoted by a red line in the above images (Fig. 2, I and J). The digital output across elements 6 in group 1 of the USAF chart appears similar for both sensors, attesting to no degradation in spatial resolution due to the PNC layer. Furthermore, the spatial resolution for our sensor under 340 nm is 3.56 lines per mm or 140 μm.

### Spectral and spatial evaluation

**Fig. 3.**
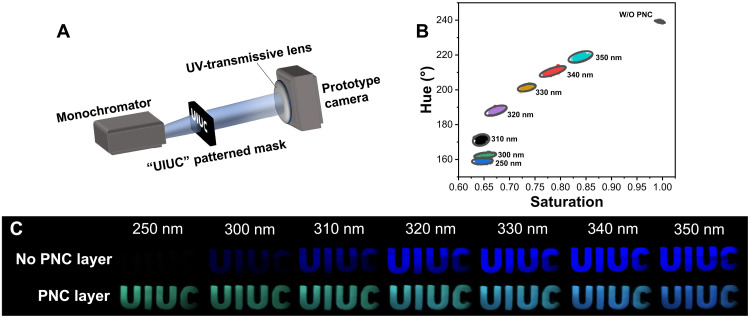
Evaluation of wavelength-resolved UV imaging capabilities. (**A**) Imaging setup schematic with a UIUC backlit mask illuminated with monochromatic light from 250 to 350 nm. (**B**) Hue-saturation spectral discrimination for diverse wavelengths. After PNC layer application, wavelengths are distinguishable with 99% confidence, a contrast to the overlap observed with the bare sensor. (**C**) Images taken with and without the PNC layer demonstrating both UV enhancement and spectral discrimination capability of the PNC-coated sensor.

To simultaneously evaluate the spectral and spatial imaging capabilities of our imaging sensor with and without the PNC layer, we imaged a back-illuminated mask containing the letters “UIUC” (Fig. 3A). The raw intensity values recorded by the three vertically stacked photodiodes are converted to hue, saturation, and value (HSV). The hue and saturation represent spectral information for the imaged target, while the value will represent intensity information. The monochromatic light was swept between 250 and 350 nm, and the hue and saturation data were obtained for the PNC-coated and original sensor (i.e., no PNC coating; Fig. 3B). The targets with different wavelengths can be distinguished with 99% confidence with our PNC-coated sensor, while the original sensor rendered the same hue and saturation outputs for all targets. In addition, a set of seven images at different illumination wavelengths were recorded (Fig. 3C). When the target was illuminated at 250 nm, only the PNC-coated imager was able to record an image of the mask. The non–PNC-coated imager was not able to capture an image because the QE is close to zero at this wavelength. For longer wavelengths, both sensors were able to capture the letters of the back-illuminated mask. However, the non–PNC-coated imager is only able to register intensity variations for the different targets. Therefore, all images appear in blue, and the intensity of the blue color varies depending on the UV photon flux.

The PNC-coated imager is able to capture more reliable images for all seven different illuminations due to the enhanced QE (Fig. 3B and fig. S3). Furthermore, the color of the images changes from green to blue as the illumination spectrum increases from 250 to 350 nm. At lower illumination wavelengths, only the PNC layer can sense these photons and produce green, fluorescent photons. As the illumination wavelengths increase, both direct photon absorption in the top photodiode and green fluorescence that is detected across all three photodiodes contribute to the photogenerated charges. Hence, the image color shifts toward blue coloration for longer wavelengths where direct photon absorption dominates the photogenerated charges.

### UV autofluorescence-based cancer identification

**Fig. 4.**
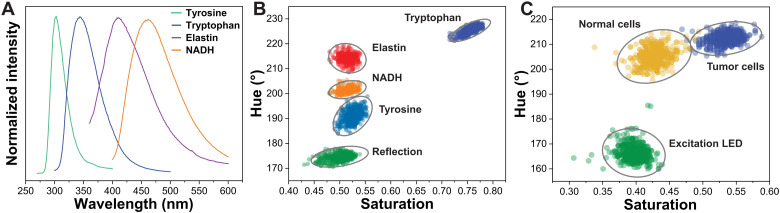
UV autofluorescence signatures and target identification with bioinspired sensor. (**A**) Distinct UV signatures from various biomolecules’ autofluorescence. (**B**) Hue-saturation representation of the autofluorescence from biomaterials, captured with our PNC-coated sensor, indicating unique target identification capability. (**C**) UV autofluorescence images of tumor and healthy cells, with respective hue-saturation measurements and confidence ellipses for each cluster.

Aromatic amino acids (e.g., tyrosine and tryptophan), proteins (e.g., elastin), and enzymes [e.g., reduced form of nicotinamide adenine dinucleotide (oxidized form) (NADH)] are present in higher concentration in cancerous tissues compared to healthy tissue due to higher metabolic activity and cell proliferation compared to normal tissue. These aromatic amino acids, proteins, and enzymes, when excited with UV light at 280 nm, exhibit distinct fluorescence spectra spanning the UV and part of the visible spectrum, i.e., 300 to 500 nm (Fig. 4A). We first evaluated our imaging device discrimination capabilities for these cancer-related biomedical materials. Four separate UV-transmissive quartz vials with solutions (or suspensions) of tyrosine, tryptophan, elastin, and NADH were excited with a 280-nm UV light source and imaged with our PNC-coated bioinspired sensor (see Materials and Methods and fig. S4). Because of the different fluorescence spectra, the color of these images varies from green for tyrosine to blue for tryptophan. The three intensity measurements per material from the top, middle, and bottom photodiodes are converted to hue-saturation representation and shown in Fig. 4B. Each material forms a distinct cluster in the hue-saturation plane despite their intensity values being the same. This experiment validates the optical optimization of our spectral imaging sensor to discriminate UV fluorescence from several cancer-related aromatic amino acids, proteins, and enzymes.

Last, we validated the ability to discriminate cancerous from normal cells based on UV autofluorescence signals recorded with our sensor. Breast cancer cell lines and normal kidney cell lines were suspended in phosphate-buffered saline (PBS) and stored in UV-transmissive quartz vials. Both vials were excited with 280-nm UV light, and the UV fluorescence was imaged from the top with our PNC-coated sensor (fig. S5). The color of the vial, which represents the autofluorescence, appears in light blue color with small observable differences between the two vials. The background appears in green color that is due to direct reflection from the 280-nm UV excitation light source. The autofluorescence intensity is higher for the cancer cells compared to normal cells (fig. S6), which is consistent with previously published results (*32*–*34*). However, these cells are also differentiable based on the spectral information represented in the hue-saturation plane (Fig. 4C). Furthermore, the spectrum of the excitation light source represents a separate cluster from the two cell lines. The wavelength-resolved imaging capabilities in the UV and visible spectrum enable our bioinspired sensor to differentiate breast cancer from normal cell lines based on the autofluorescence of endogenous fluorophores.

## DISCUSSION

In summary, we have developed a bioinspired imaging sensor for wavelength-resolved UV imaging and label-free biomarker detection based on the use of vertically stacked photodiodes and highly fluorescent PNCs. Similar to the UV-sensitive visual system of the *P. xuthus* butterfly, our complementary metal-oxide semiconductor (CMOS) camera is based on the simultaneous detection of both a visible fluorescence signal (generated by the PNC layer) and a portion of the original UV signal that is rapidly attenuated and detected by the top silicon layer. Digital processing of the sensor’s RGB signals allows rapid mapping and identification of the UV signatures.

The cutting-edge UV imaging technologies now use backside-illuminated silicon detectors and excel in achieving high QE across both UVB and UVA spectra. However, no existing imaging technology can carry out wavelength-resolved UV imaging with high spatial resolution and in real time. Our bioinspired imaging approach integrates PNCs with vertically stacked silicon photodetectors, facilitating wavelength-resolved UV imaging with high spatial and temporal resolution.

This technology is likely to have broad applications, especially for medical imaging, military target tracking, and industrial automation. Label-free UV fluorescence imaging data from aromatic amino acids and cancer/normal cells have allowed real-time imaging and wavelength differentiation of the intrinsic UV signatures of these biomedical materials at approximately 99% confidence. Furthermore, we envision that two-color nanocrystals with distinct UV absorption curves could be used in a tandem or mixed configuration for converting any UV light into two-color fluorescence signals, likely resulting in an improvement in wavelength separation of UV signatures.

## MATERIALS AND METHODS

### Synthesis of the cesium-oleate precursor

A 1.3-g Cs_2_CO_3_ (Thermo Fisher Scientific, United States) was loaded into a 50-ml three-neck round-bottom flask with 4 ml of oleic acid (OA; Thermo Fisher Scientific, USA) and 16 ml of 1-octadecene (ODE; Sigma-Aldrich, USA). The system was then degassed for 10 min and dried under vigorous stirring and vacuum conditions for 1 hour at 120°C. Afterward, the mixture was heated to 150°C under nitrogen atmosphere until all Cs_2_CO_3_ reacted and the solution became clear. Cs-oleate precursor must be heated to 120°C before use because it precipitates at room temperature.

### Synthesis of CsPbBr_3_ PNCs

A 2 mmol PbBr_2_ (Thermo Fisher Scientific, USA), 5 ml of OA, 5 ml of oleylamine (Sigma-Aldrich, USA), and 7.5 ml of ODE were loaded into a 50-ml three-neck round-bottom flask. The system was dried under vacuum for 1 hour at 120°C to remove the moisture from the raw materials. The temperature was then elevated to 180°C under nitrogen atmosphere and kept at this temperature for 2 min. The Cs-oleate precursor (2 ml, 0.4 M) was then quickly injected under vigorous stirring. After 5 to 10 s, the reaction mixture was quenched in an ice bath. The crude nanocrystal solution with bright green fluorescence under UV light was centrifuged at 8500 rpm for 10 min to discard the supernatant containing unreacted precursors. The precipitate was collected to fabricate the down-conversion layer.

### Fabrication of PNC down-conversion layer

The as-synthesized CsPbBr_3_ PNCs were dissolved into hexane (Sigma-Aldrich, USA) with a weight percentage of 30 wt %. The solution was then spin-coated directly onto the top of the CMOS imaging sensor (Foveon X3 F13 14.4 Megapixel CMOS Direct Image Sensor) at 1500 rpm for 60 s. After that, the precoated device was retained on a heating plate at 50°C under a fume hood for another 15 min to remove residual solvents.

### Material characterization

Cross-sectional SEM image was captured by the Thermo Scios2 Dual-Beam SEM/FIB (Thermo Fisher Scientific, USA). TEM image of the PNCs was taken by the FEI Tecnai G2 F20 S-TWIN STEM system (Thermo Fisher Scientific, USA). XRD was measured by the D8 Advance XRD System (Bruker Corporation, USA). The absorbance spectrum was measured by a Genesys 10S UV-VIS spectrophotometer (Thermo Fisher Scientific, USA). The PL emission spectrum was acquired by the RF-6000 spectro Fluorophotometer (Shimadzu Corporation, Japan). The absolute PLQY was determined using a fluorescence spectrometer (Horiba PTI QuantaMaster 400 steady-state fluorescence system) with an integrated sphere (C9920-02, Hamamatsu Photonics, Japan) excited by an LED light source (F5 DIP LED, 3 V, 20 mA) at a wavelength of 450 nm.

### Device characterization

Absorption coefficient of our PNCs is calculated by α = *A*/*h*, where α is the absorption coefficient; *A* is the absorbance, which is measured on PNC thin films coated on quartz glasses with the Varian Cary 5G spectrometer (Agilent Technologies, USA); and *h* is the thickness of the PNC films measured by profilometry analysis with the VK-X1000 Laser Scanning Microscope (Keyence Corporation, USA). In linearity measurements, the camera outputs were acquired with an integrating sphere (58-585, Edmund Optics, USA), averaged from 100 × 100 pixels at the center of the imager over 25 frames. The light sources were from the Mounted LED Series of Thorlabs Inc. (M310L1, M340L5, M385L3, M470L5, M530L4, and M625L4), and the incident optical power intensities were measured at the same place as the imager by a PM100D power meter console (ThorLabs Inc., USA) with a S130VC photodiode sensor (Thorlabs Inc., USA). In FPN measurements, uniform plane wave was generated from an integrating sphere (58-585, Edmund Optics, USA). The light source was a 385-nm UV LED (M385L3, Thorlabs Inc., USA), and the incident light intensity was controlled to generate outputs at half the linear full-scale range. Then, the outputs of 100 × 100 pixels at the center of the imager were counted to generate the histograms, and the SD of these outputs was the FPN. In QE measurements, almost the same measurement scheme as in linearity measurements was used, except that the light source was the monochromator incorporated inside the RF-6000 spectro Fluorophotometer (Shimadzu Corporation, Japan) with a 150-W Xenon lamp. In HSV measurements, areas of interest (AOIs) with size of 25 × 25 pixels were picked, and the RGB output of each pixel inside the AOIs was converted to HSV to generate the hue-saturation scatter plots. During UV imaging processes, a UV-transmissive quartz lens (UV2528B, Industrial Camera Sales) was used on the camera.

### Cell culture

The human lung cancer cell line A549 and normal kidney cell line human embryonic kidney–293 were purchased from the American Type Culture Collection (USA). These cells were cultured in Dulbecco’s modified Eagle’s medium supplemented with 10% fetal bovine serum and 1% penicillin-streptomycin at 37°C with 5% CO_2_. At 80% confluency, the cells were treated with 0.05% trypsin-EDTA (Thermo Fisher Scientific, USA), transferred to 50-ml conical tubes with 5 ml of culture medium, and centrifuged at 300 rpm for 3 min to discard the supernatant. Subsequently, the cell pellets were resuspended in 2 ml of PBS at 5 million cells/ml in triplicate. Last, the cells were distributed to UV-transmissive cuvettes for imaging.

### Optimization of PNC layer thickness

To optimize the PNC layer thickness for wavelength-resolved UV sensing, we conducted a model-based analysis to evaluate the photocurrent generated in the top, middle, and bottom photodiodes resulting from fluorescence emitted by the PNC layer and direct photon absorption. The cross-sectional diagram of the imaging sensor, depicted in Fig. 1E, showcases the vertically stacked photodiodes composed of silicon p-n junctions. Under the assumption of the depletion approximation, each individual p-n junction can be divided into three distinct regions: the quasi-neutral n-region, the depletion region, and the quasi-neutral p region (as illustrated in fig. S1).

We denote the top and bottom edges of the depletion region as *x*_1_ and *x*_2_, respectively. The thickness of the depletion region (i.e., *x*_1_ and *x*_2_ distance) is influenced by the reverse bias voltage and the doping levels of both the p and n regions. Furthermore, the thickness of the entire photodiode is represented by *x*_3_. According to the depletion approximation, the photocurrent density resulting from the generation of electron-hole pairs within the space-charged depletion region can be expressed as

$$j_{ph}^{d}(\lambda )=qF_{0}(e^{-\alpha x_{1}}-e^{-\alpha x_{2}})\;[A/cm^{2}]$$ (1)

In Eq. 1, *q* is the electron charge, *F*_0_ is the incident photon flux, and α is the absorption coefficient of silicon at wavelength λ. In the quasi-neutral regions, where no electric field is present, only diffusion current is generated. The diffusion current density generated within the quasi-neutral n region and quasi-neutral p region can be represented by Eqs. 2 and 3, respectively

$$j_{ph}^{n}(\lambda )=\frac{qF_{0}}{\alpha x_{1}}[1-(\alpha x_{1}+1)e^{-\alpha x_{1}}]\;[A/cm^{2}]$$ (2)

$$j_{ph}^{p}(\lambda )=\frac{qF_{0}}{\alpha (x_{3}-x_{2})}\{[\alpha (x_{3}-x_{2})-1]e^{-\alpha x_{2}}+e^{-\alpha x_{3}}\}\;[A/cm^{2}]$$ (3)

The spectral response η, which is the fraction of incident photons contributing to generation of photocurrent as a function of wavelength λ, can be derived by adding the photocurrents in all three regions

$$\eta (\lambda )=\frac{j_{ph}^{d}(\lambda )+j_{ph}^{n}(\lambda )+j_{ph}^{p}(\lambda )}{qF_{0}}=\frac{1}{\alpha }\left(\frac{1-e^{-\alpha x_{1}}}{x_{1}}-\frac{e^{-\alpha x_{2}}-e^{-\alpha x_{3}}}{x_{3}-x_{2}}\right)\;[\mathrm{electrons}/\mathrm{photons}]$$ (4)

Assuming that the conversion gain of all photodiodes is independent of the incident wavelength, the digital outputs of the red, green, and blue channels are directly proportional to the number of electron-hole pairs generated within the bottom, middle, and top photodiodes, respectively. These quantities are determined by the spectral responses, η, of the three vertically stacked photodiodes and the number of incident photons. The incident photon flux impinging on each photodiode follows an exponential characteristic with respect to its depth. Therefore, the ratio between the digital outputs of red, green, and blue channels (R, G, and B, respectively) is

$$R:G:B=\eta_{R}(\lambda )e^{-\alpha (d_{B}+d_{G})}:\eta_{G}(\lambda )e^{-\alpha d_{B}}:\eta_{B}(\lambda )$$ (5)

In Eq. 5, the variables *d*_B_ and *d*_G_ represent the thicknesses of the top and middle photodiodes, respectively. The variables η_R_, η_G_, and η_B_ represent the spectral responses of the bottom, middle, and top photodiodes, respectively. By obtaining the ratio of the RGB values, it is possible to calculate the hue and saturation data corresponding to each RGB value.

Assuming that our PNC-coated sensor is exposed to monochromatic light, simulations can be conducted to analyze the hue of the sensor’s digital output, considering the illumination wavelength and the thickness of the PNC layer. The PNC layer thickness substantialy influences the ratio of UV and visible photons reaching the photodiodes. As illustrated in Fig. 1H, a thicker PNC layer enhances the absorption and conversion of incident UV light, resulting in a higher proportion of fluorescent green photons reaching the photodiodes. Conversely, a thinner PNC layer allows more UV light to pass through, leading to a greater number of UV photons reaching the photodiodes. However, in both cases, the UV spectral discrimination capabilities of our sensor are compromised. The optimum separation and highest spectral discrimination in the hue value for different incident photons are achieved when the PNC layer thickness is set to 2 μm.
