# Supplementary material for: Bioinspired, vertically stacked, and perovskite nanocrystal–enhanced CMOS imaging sensors for resolving UV spectral signatures
Source: Sci Adv. 2023 Nov 3;9(44):eadk3860. doi: 10.1126/sciadv.adk3860 (PMC10624339; doi:10.1126/sciadv.adk3860)
Supplement: Supplementary file 1 — Figs. S1 to S6 [file sciadv.adk3860_sm.pdf]

Science Advances
AAAS

Supplementary Materials for

Bioinspired, vertically stacked, and perovskite nanocrystal–enhanced CMOS imaging sensors for resolving UV spectral signatures

Cheng Chen *et al.*

Corresponding author: Shuming Nie, nies@illinois.edu; Viktor Gruev, vgruev@illinois.edu

Sci. Adv. **9**, eadk3860 (2023)
DOI: 10.1126/sciadv.adk3860

This PDF file includes:

Figs. S1 to S6

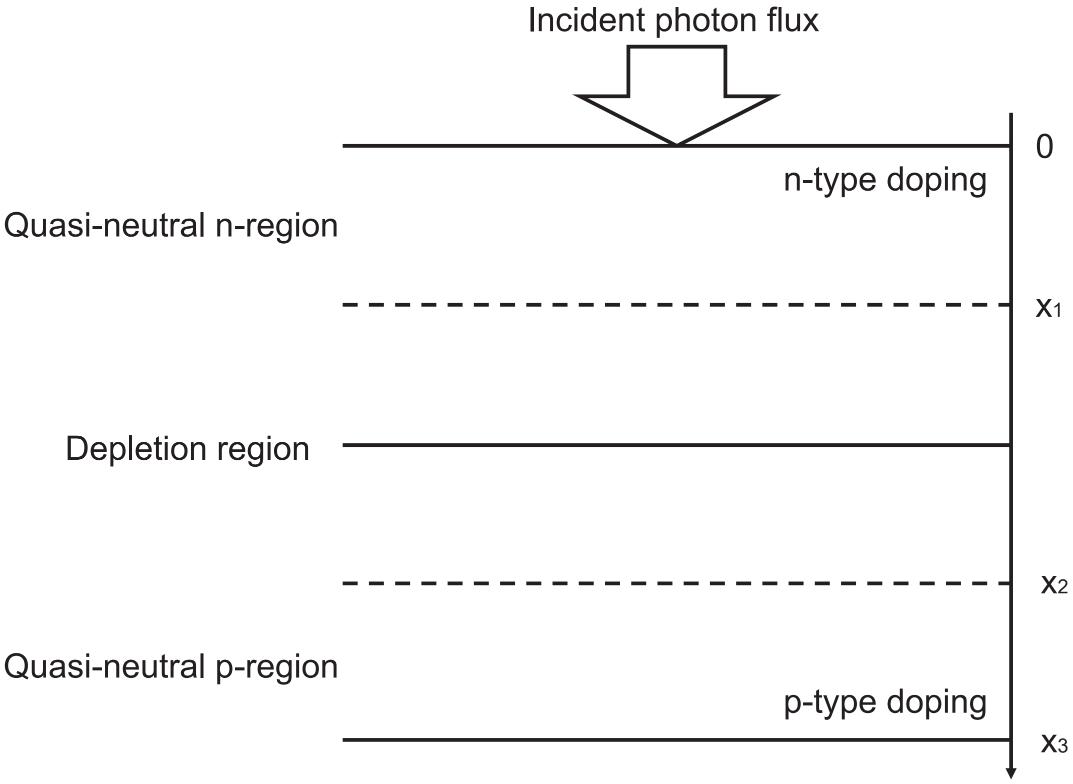

Fig. S1. Silicon P-N junction cross profile. Schematic of different regions in the depletion approximation for the P-N junction of the photodiodes

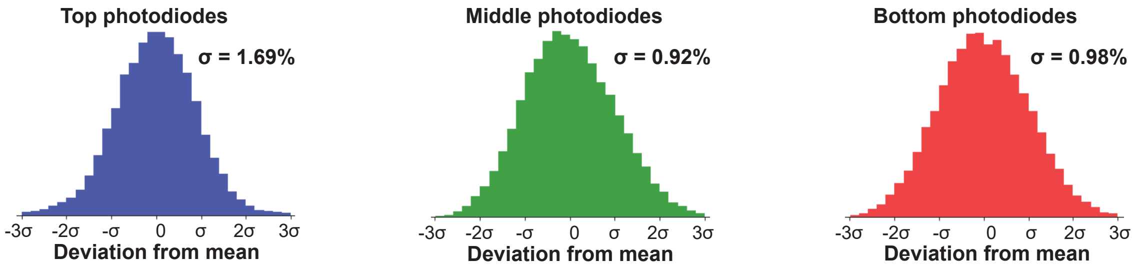

Fig. S2. FPN evaluation of our sensor. FPN histograms at half the linear full-scale range for the top, middle, and bottom photodiodes. The FPN is the standard deviation σ of the histograms. The spatial uniformity for the three vertically stacked photodetectors with the PNC top layer are 1.69%, 0.92%, and 0.98%, respectively.

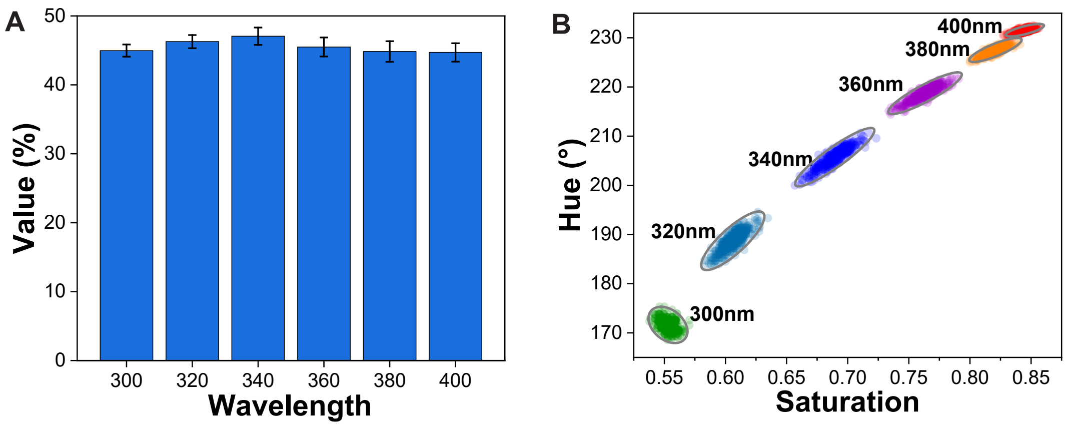

Fig. S3. Spectral discrimination in the UV spectrum. Intensity (**A**) and hue-saturation spectral discrimination (**B**) of narrow-band monochromatic targets from 300 nm to 400 nm. Although the intensity for these targets are matched, the hue-saturation clusters indicate spectral discrimination of our sensor in the UV spectrum.

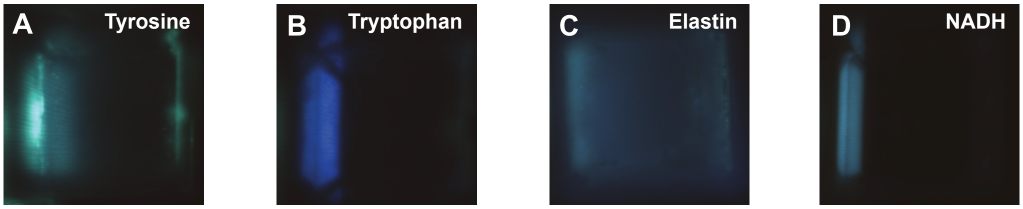

Fig. S4. UV autofluoresence of various biomolecules. UV autofluoresence under 280nm excitation light, captured with our PNC-coated bioinspired sensors of 20 mg/mL solutions or suspensions of (**A**) tyrosine, (**B**) tryptophan, (**C**) elastin, and (**D**) NADH. The fluorescence of these biomedical materials exhibit different colors with our camera.

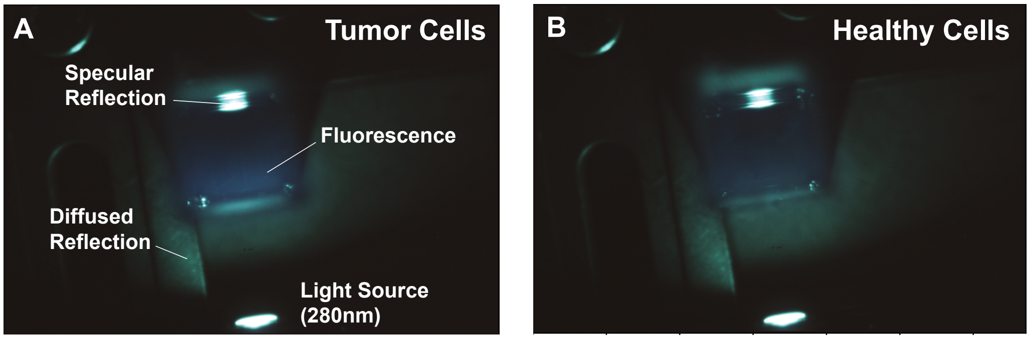

Fig. S5. UV autofluoresence images of tumor and healthy cells in PBS. The (**A**) tumor and (**B**) healthy cells are excited with 280 nm LED illumination from the side and imaged from the top with our PNC-coated spectral sensor. The autofluorescence appears in light blue color with small observable differences between the two vials. The background appears in green color which is due to direct reflection from the 280 nm UV excitation light source.

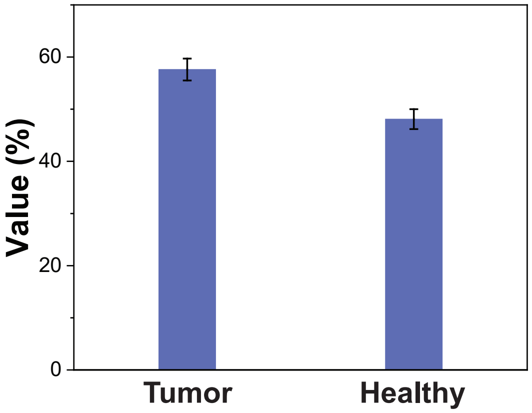

Fig. S6. Intensity measurements of tumor and healthy cell UV autofluorescence. The autofluorescence intensity is higher for the cancer cells compared to normal cells, which is consistent with previously published results.
